# Retroperitoneoscopic approach for highly complex posterior renal hilar tumors

**DOI:** 10.1590/S1677-5538.IBJU.2019.0074

**Published:** 2020-02-20

**Authors:** Jose Luis Bauza, Valentí Tubau, Jorge Guimerà, Luis Ladaria, Carlos Aliaga, Pedro Piza, Enrique Pieras

**Affiliations:** 1 Hospital Universitario Son Espases Department of Urology Palma de MallorcaIlles Balears Spain Department of Urology, Hospital Universitario Son Espases, Palma de Mallorca, Illes Balears, Spain

## Abstract

**Objectives:**

To show our single-center experience in retroperitoneoscopic approach for highly complex posterior hilar tumors. Minimally invasive nephron sparing surgery for renal hilar tumors is extremely challenging due to their anatomic location, close to the main renal vessels and the collecting system (1). Transperitoneal approach is feasible, but highly complex because the anterior disposition of the vasculature. Retroperitoneal approach can easily provide access to the posterior hilar structures and the posterolateral surface of the kidney(2, 3).

**Materials and Methods:**

We retrospectively reviewed our hilar renal tumor database and analyzed those in which a retroperitoneoscopic approach was chosen. The RENAL score was then calculated, and operative and ischemia times were recorded. We also collected the mean hospital stay and the presence of complications. Pathology reports and follow-up were also gathered.

**Results:**

Five of our twelve highly complex hilar renal tumor patients were treated using a retroperitoneoscopic approach. Mean RENAL score was 10. Mean operative time was 135 minutes. Mean warm ischemia time was 14 minutes. Mean hospital stay was 4 days. We have recorded 2 complications. One patient required a transfusion and another presented with an urinary fistula which was treated by double J stent placement. The pathology report showed a clear cell renal cell carcinoma pT1a in most of the cases. Only one patient had a positive margin. To date, no recurrences have been noticed.

**Conclusions:**

The treatment of complex renal hilar tumors in a minimally invasive fashion is highly challenging even in experienced hands. Retroperitoneal partial nephrectomy is feasible, safe and effective for the treatment of such lesions. Long-term oncologic outcomes of this approach are awaited.
